# Absence of *Plasmodium falciparum* Histidine-Rich Protein 2 and 3 (Pfhrp2/3) Gene Deletions in Rizal, Palawan, the Philippines

**DOI:** 10.4269/ajtmh.25-0616

**Published:** 2026-09-22

**Authors:** Jennifer S. Luchavez, Alison Paolo N. Bareng, Freya Spencer, Samuel G. Thorburn, Maria Lourdes M. Macalinao, Fe Esperanza J. Espino, Chris J. Drakeley, Julius Clemence R. Hafalla, Lynn Grignard

**Affiliations:** ^1^ Department of Parasitology, Research Institute for Tropical Medicine, Muntinlupa, Philippines;; ^2^ Infection and Global Health Division, Walter and Eliza Hall Institute, Melbourne, Australia;; ^3^ Faculty of Infectious and Tropical Diseases, Department of Infection Biology, London School of Hygiene and Tropical Medicine (LSHTM), London, United Kingdom

## Abstract

Microscopy and rapid diagnostic tests (RDTs) are used in the Philippines as the gold standard for malaria diagnosis. However, parasites evading RDT detection have recently emerged in other settings as a result of deletions in *Plasmodium falciparum* histidine-rich protein genes (*pfhrp2* and *pfhrp3*). Failure to detect and treat individuals carrying these deletions may result in increased malaria transmission and hinder elimination efforts. In addition to the implications for malaria elimination efforts, we emphasize that false-negative RDT results caused by *pfhrp2/3* deletions pose a critical risk to patient outcomes, as individuals with active *P. falciparum* infections may go untreated. Sixty-three samples collected from Palawan, the Philippines, were interrogated for the presence of *Plasmodium* isolates and *pfhrp* deletions. All 63 samples were microscopically positive for *P. falciparum*. Although only 27 (43%) were positive for RDTs, all samples were confirmed free of *pfhrp2* and *pfhrp3* deletions by nested polymerase chain reaction (PCR) tests. RDT false negatives in the study were most likely caused by low parasite densities. Incorporating molecular monitoring of histidine-rich protein 2 deletions into routine malaria surveillance would improve assessments of the performance of the current RDTs and help strengthen strategies toward malaria elimination by 2030.

## INTRODUCTION

The Philippines is committed to achieving malaria-free status by 2030.^1^ The country achieved a 92% decrease in malaria cases and a 98% decrease in malaria deaths from 2004 to 2021, with Palawan being the only province to report local cases since 2022. Cases in Palawan increased between 2023 and 2024, which is likely due in part to improved surveillance.^2^ Malaria surveillance in low-transmission settings relies on sensitive diagnostics, including asymptomatic infections that may infect mosquitoes and sustain transmission. Detecting and eliminating all parasites are paramount to attaining malaria-free status in the Philippines and beyond, eventually.

The Philippines employs microscopy as the gold standard for the diagnosis of malaria and uses *Plasmodium falciparum*/*pan Plasmodium* dual-antigen rapid diagnostic tests (RDTs) in remote areas.^3^ These tests target *P. falciparum* histidine-rich protein 2 (PfHRP2) and additionally either lactate dehydrogenase (pLDH) or aldolase. PfHRP2 is expressed exclusively in P. falciparum, whereas pLDH and aldolase are found in all Plasmodium species.^4^

Despite their numerous advantages, concerns have been raised about the sensitivity of RDTs. An increasing number of studies have recently reported false-negative RDT results due to parasites harboring deletions of the *pfhrp2* gene. PfHRP2 is a highly expressed protein, and it is the most frequent target of commercially available RDTs. Failure to detect and treat individuals carrying *pfhrp2* gene deletions could lead to an increased transmission of malaria and the selection of PfHRP-deleted parasites.^5^^–^^8^ Such a scenario poses a threat to national malaria control programs and the malaria elimination agenda.

Here, we determined whether *P. falciparum* isolates from the Philippines carry deletions in the *pfhrp2* gene and in a related gene, *pfhrp3*. Immunological cross-reactivity between HRP2 and HRP3 could mask *pfhrp2* deletions by recognizing HRP3 protein expression. Samples were obtained from a larger cross-sectional survey in Palawan, the Philippines, conducted from June 2016 to May 2017, that aimed to enhance malaria surveillance for elimination efforts by sampling dried blood spots (DBS) in health facility attendees and their companions.^9^ DNA was extracted from dried blood spots (DBS) and analyzed for the prevalence, diversity, and species of parasites, as well as the presence of the *pfhrp2* and *pfhrp3* genes.

Ethical clearance was obtained from the RITM and LSHTM institutional review boards (RITM 2016-04 and LSHTM IRB No. 11597). All participants provided and signed informed consent and assent as required. Samples were collected as part of a rolling cross-sectional survey of parasite prevalence and seroprevalence among health facility attendees in the municipality of Rizal, Palawan province.^9^^,^^10^ Finger-prick blood samples were used to diagnose malaria by microscopy and dual-antigen RDT (SD Bioline Malaria Ag Pf/Pv and SD Bioline Malaria Ag Pf/Pan; Abbott, Abbot Park, Illinois, USA).

Sixty-three samples (27 RDT-positive and 36 RDT-negative samples) were randomly selected for molecular analysis at the LSHTM. DNA was extracted using the QIAamp DNA mini kit with three blood spot punches (3 mm in size each) and eluted in 100 *µ*L of elution buffer (Qiagen; Hilden, Germany). Samples were tested in duplicate using a conventional two-step nested polymerase chain reaction (PCR) assay targeting the *Plasmodium*-specific small subunit ribosomal RNA (*18SSU rRNA*) gene to detect the presence of current pan-genus malaria infections.^11^ Further, the samples were analyzed targeting the single-copy merozoite surface protein 2 (*msp2*) gene. The products of the *msp2* PCR reactions were analyzed by capillary electrophoresis length polymorphism genotyping^12^ to determine the multiplicity of infection (MOI). *Pfhrp2* and *pfhrp3* exon 2 were amplified in triplicate to look for gene deletions using agarose gel, following previously published methods.^13^ In addition, samples were amplified in a pan *Plasmodium 18S* and human *β2M* quantitative polymerase chain reaction (qPCR)^14^ to determine parasite density and sample integrity, respectively, alongside a serial dilution of the WHO international standard (4.7 × 10^5^ parasites/*µ*L − 4.7 × 10^−1^ parasites/*µ*L). Figure 1 shows a flow chart of the molecular analysis performed in this study.

**Figure 1.**
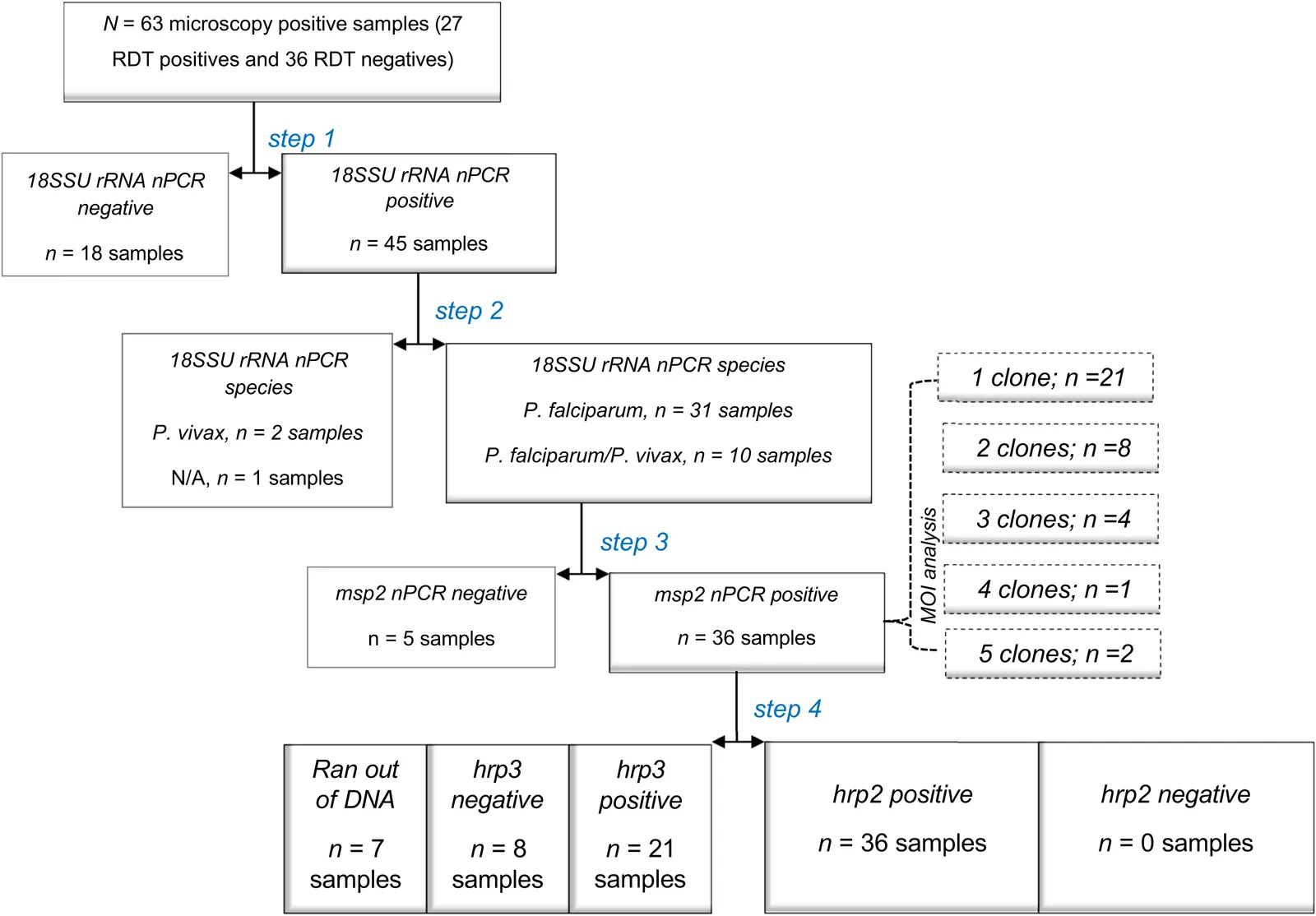
Flow chart of molecular analysis and MOI. All samples (*n* = 63) were analyzed in a stepwise manner to determine the presence of true *pfhrp* deletions. In step 1, 63 samples were analyzed by *18SSU rRNA* nPCR to test for the presence of pan-*Plasmodium.* Positive samples (*n* = 45) were taken forward and speciated by nPCR in step 2. Only *Plasmodium falciparum* monoinfections and *P. falciparum/P. vivax* mixed infections were brought forward in step 3. In step 3, samples were amplified by *msp2* nPCR to test for the ability to amplify single-copy genes. Only samples that amplified in the single-copy gene PCR were taken forward and amplified in the *hrp* nPCRs in step 4. MOI, the number of parasite clones in each *msp2* positive sample (*n* = 36), is shown. The median and IQR = 1 (IQR: 1–2). IQR = interquartile range; MOI = multiplicity of infection; nPCR = nested polymerase chain reaction; PCR = polymerase chain reaction.

Data were analyzed using GraphPad Prism (GraphPad Prism Software, LLC; San Diego, CA, USA). Parasite densities were expressed as geometric mean log_10_ parasite densities and 95% confidence intervals (CI). An unpaired *t*-test was used to compare the parasite densities in RDT-positive and RDT-negative samples. *P* <0.05 was regarded as statistically significant. All 63 selected samples were positive by microscopy: 27 (43%) were RDT-positive and 36 (57%) RDT-negative. To interrogate the samples for *pfhrp* deletions, we followed a stepwise molecular detection approach (Figure 1).

First, we tested for the presence of pan-*Plasmodium*. The *18SSU rRNA* amplification was successful in 45 (*n* = 45/63, 71%) samples, which were further analyzed in species-specific reactions. Two samples were not speciated (2/45, 4%), and two samples (2/45, 4%) had *P. vivax* monoinfections and were excluded from further analysis. Of the 41 samples remaining, 10 (22%) were dual infections with *P*. *vivax*, and 31 (69%) were *P. falciparum* monoinfections.

To test for single-gene copy amplification, the 41 remaining samples were further analyzed by *merozoite surface protein 2* (*msp2*) PCR. Thirty-six samples (87.8%) were *msp2*-positive; the *msp2*-negative samples were excluded from further analysis. *Msp2-*positive samples had a median MOI of 1 (interquartile range [IQR]: 1–2) with a maximum number of five clones found (Figure 1).

Next, the remaining 36 samples were tested for the presence of the *pfhrp* genes. All samples (*n* = 36), irrespective of their corresponding RDT result, were *pfhrp2* positive by nPCR. The samples were also tested for the presence of the *pfhrp3* gene (*n* = 29); seven samples were excluded from *pfhrp3* amplification and further analysis as DNA had run out. Twenty-one samples (72.4%) were *pfhrp3* positive. Eight samples failed amplifications (27.6%) in the *pfhrp3* nPCR, seven of which were RDT-negative. Three RDT-negative and *pfhrp3*-negative samples (42.9%, 3/7) were mixed *P. falciparum*/*P. vivax* infections.

To explain the discrepant *pfhrp* PCR results and RDT results, the relationship between parasite densities and RDT outcomes was further explored (Figure 2). The geometric mean parasite density by microscopy was 29,697.7 (95% CI: 8,950–98,545) and 627.9 (95% CI: 147.1–2,680) parasites/*µ*L for RDT-positive and RDT-negative samples, respectively. The geometric mean parasite density by qPCR was 62,951.1 (95% CI: 19,581.5–202,377) and 918.1 (95% CI: 191.3–4,405.3) parasites/*µ*L for RDT-positive and RDT-negative samples, respectively. The *pfhrp3* negative samples had the lowest parasite density of all samples by both microscopy and *18S* qPCR (dashed boxes, Figure 2). All but one of the *pfhrp3*-negative samples were RDT-negative (Figure 2). The results from microscopy and qPCR confirmed lower parasite density in RDT-negative/*pfhrp2*-positive samples in comparison with RDT-positive/*pfhrp2*–positive samples.

**Figure 2.**
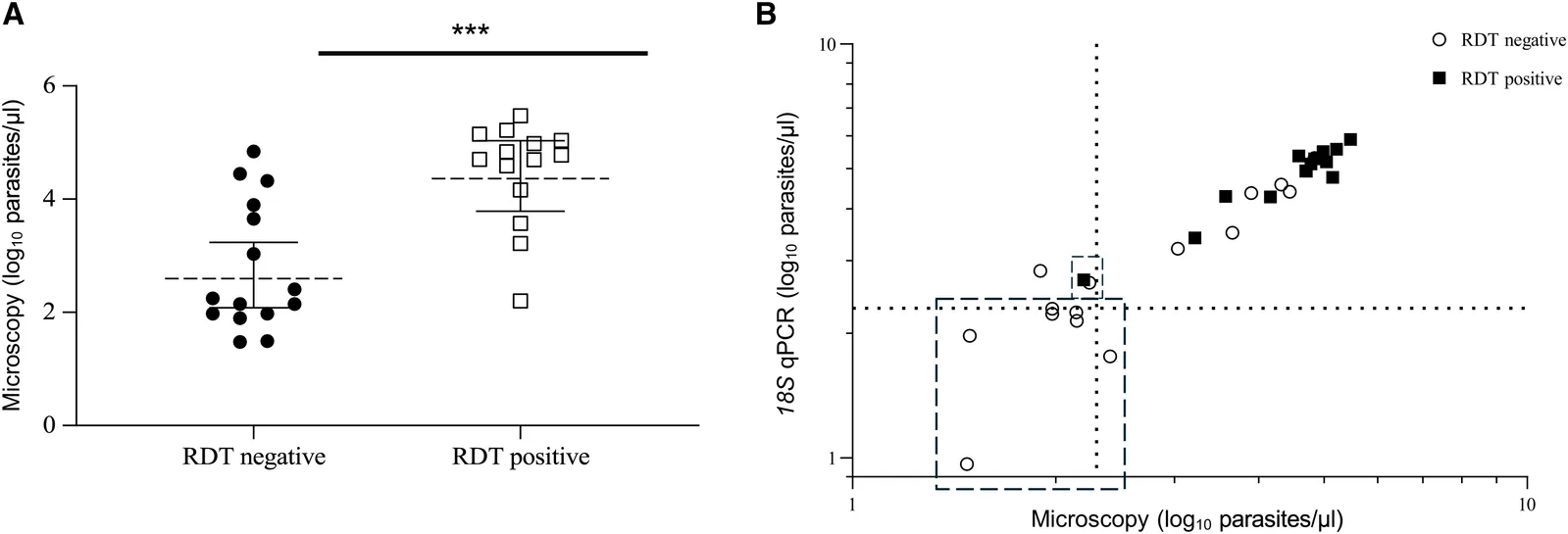
Measurements of *Plasmodium falciparum* densities and RDT outcomes. Parasite density in RDT and *pfhrp3*-positive and negative samples. (**A**) Parasite density by microscopy in RDT-positive and RDT-negative samples. Dashed line, geometric mean log_10_ parasite density, and 95% CI; *** unpaired *t*-test, *t* = 4.371; df = 27; *P* = 0.0002. (**B**) Microscopy versus qPCR in RDT-positive and RDT-negative samples. Dotted lines represent 200 parasites/*µ*L, a standard RDT detection threshold. The dashed box contains all *pfhrp3* negative samples. CI = confidence interval; df = degrees of freedom; qPCR = quantitative polymerase chain reaction; RDT = rapid diagnostic test.

This is the first study investigating *pfhrp* deletions in the Philippines. The detection of gene deletions, where the absence of amplification indicates a deletion, requires considerable assay scrutiny.^8^ All samples were confirmed free of *pfhrp2* deletions. *Pfhrp3* deletions were also assessed in the event of detecting any RDT-positive but *pfhrp2-*negative samples. Eight samples were *pfhrp3*-negative by nPCR. The deletion of *pfhrp3* in *pfhrp2*-positive parasites is of limited importance, because the lack of HRP3 expression is unlikely to impact HRP2-based RDT results in the presence of HRP2 protein.^7^^,^^13^ The *pfhrp3*-negative samples had low overall DNA content as shown by qPCRs targeting parasite (*18S*) and human genes (*β2M*), suggesting that the eight *pfhrp3*-negative samples failed to amplify because of insufficient DNA rather than *pfhrp3* gene deletions.^15^^,^^16^ We cannot exclude the possibility of masked *pfhrp* deletions in multiclonal samples. The impact of minor clones harboring deletions on surveillance is negligible in the presence of the majority of clones still detected by RDTs.

In addition to gene deletions, false-negative RDTs are caused by several factors: operator efficiency, inadequate transport or storage, and parasite density.^7^^,^^16^ The dual RDTs used here detect as low as 50 parasites/*µ*L,^17^ but sensitivity decreases to <81% at parasite densities below 200 parasites/*µ*L.^18^ Over half of the RDT-negative samples in this study had a parasite density of less than 200 parasites/*µ*L. Very high parasite densities can also cause false-negative RDT results due to the prozone effect (>300,000 parasites/*µ*L), which was not found in the current study.^19^

The current study had several limitations. First, the number of samples under investigation was limited. Second, the nested PCR amplifications are labor-intensive and high volumes of sample are needed to complete all the reactions and repeats. Third, the ability of each assay to detect and confirm *pfhrp* deletions in field samples varies, despite a limit of detection of ∼1 parasite/*µ*L for all assays on the WHO international standard. This variability in detection is especially true at low parasite densities near the limit of detection, because the stochastic nature of PCR means that samples cannot always be reproducibly amplified, making data interpretation difficult.

The WHO recommends switching to non-HRP2–based tests in areas where 5% or more of the test population lacks the HRP2 protein. Currently, the diagnostic policy in the Philippines for malaria detection is to use either microscopy or dual-antigen RDTs in hard-to-reach areas. To maximize the use of either traditional HRP-based or ultra-sensitive HRP-RDTs, further investigation of *pfhrp* deletions in the Philippines is warranted. A larger sample set should be analyzed using a multiplex qPCR assay to probe for gene deletions.
